# Thiamine Use in Hospitalized Patients: A Clinical Review

**DOI:** 10.1155/jnme/7143046

**Published:** 2026-02-11

**Authors:** Rowan E. Rosewarne, Nicholas Farina

**Affiliations:** ^1^ Department of Pharmacy Services, University of Michigan Health 1500 E Medical Center Dr, Ann Arbor, Michigan, 48109, USA; ^2^ Department of Pharmacy Services, University of New Mexico Hospital 2211 Lomas Blvd NE, Albuquerque, New Mexico, 87106, USA

## Abstract

Thiamine, or vitamin B1, is a water‐soluble vitamin necessary for multiple metabolic processes throughout the human body. Thiamine is primarily obtained from our diet and is found in sources such as whole grains, legumes, and pork. Short bowel syndrome, severe malnutrition, alcohol use disorder, or diuretic use can all lead to thiamine deficiency. Thiamine deficiency may contribute to significant morbidity if not promptly identified and treated. Thiamine supplementation has been established as the primary treatment for patients with Wernicke’s encephalopathy. Supplementation of thiamine is also commonly provided to patients at risk for refeeding syndrome to prevent exacerbation of an underlying thiamine deficiency when nutrition is reintroduced. More recent studies have investigated thiamine supplementation for a broader use in patients with sepsis, impaired lactate clearance, and delirium. The purpose of this review is to provide an overview of the indications for thiamine supplementation in hospitalized patients, analyze literature for historical and emerging thiamine supplementation utilization, and provide a framework for supplementation based on indication.

## 1. Introduction

Thiamine, or vitamin B1, is a water‐soluble vitamin essential for human health and is utilized in glycolysis, the Krebs cycle, and the pentose phosphate pathway. Thiamine can be obtained from many diverse sources like food products, nutritional products, and some dietary supplements (Table 1) [1–5]. The recommended dietary allowance for thiamine is dependent on age and sex. The recommendation for adult men, adult women, and women during pregnancy and lactation is 1.1, 1.2, and 1.4 mg per day, respectively [6].

**TABLE 1:** Sources of thiamine.

Multivitamin	1–2 mg^†^
Enteral nutrition	0.41–1.2 mg/250 mL^†^
Intravenous thiamine	200 mg/2 mL
Oral thiamine	Tablets: 50 mg, 100 mg, 250 mg Liquid: 25 mg/mL

*Dietary Sources*	
Breakfast cereals, fortified with 100% of the DV for thiamin, 1 serving	1.2 mg
Egg noodles, enriched, cooked, 1 cup	0.5 mg
Pork chop, bone in, broiled, 3 ounces	0.4 mg
Trout, cooked, dry heat, 3 ounces	0.4 mg
Black beans, boiled, ½ cup	0.4 mg
English muffin, plain, enriched, 1	0.3 mg
Mussels, blue, cooked, moist heat, 3 ounces	0.3 mg
Tuna, bluefin, cooked, dry heat, 3 ounces	0.2 mg
Macaroni, whole wheat, cooked, 1 cup	0.2 mg
Acorn squash, cubed, baked, ½ cup	0.2 mg
Rice, brown, long grain, not enriched, cooked, ½ cup	0.2 mg
Rice, white, long grain, enriched, cooked, ½ cup	0.1 mg
Bread, whole wheat, 1 slice	0.1 mg
Orange juice, prepared from concentrate, 1 cup	0.1 mg
Sunflower seeds, toasted, 1 ounce	0.1 mg
Beef steak, bottom round, trimmed of fat, braised, 3 ounces	0.1 mg
Yogurt, plain, low fat, 1 cup	0.1 mg
Oatmeal, regular and quick, unenriched, cooked with water, ½ cup	0.1 mg
Corn, yellow, boiled, 1 medium ear	0.1 mg
Milk, 2%, 1 cup	0.1 mg
Barley, pearled, cooked, 1 cup	0.1 mg

^†^Thiamine content dependent on the specific product or formula chosen. Consult with the manufacturer label.

**FIGURE 1:**
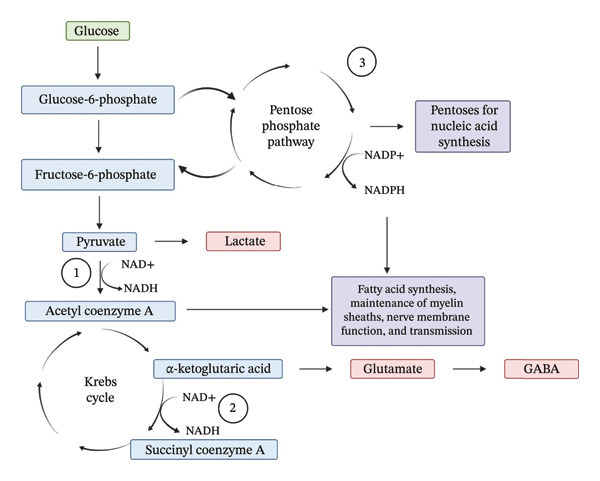
Thiamine use in metabolic pathways. NAD+, oxidized nicotinamide adenine dinucleotide; NADH, reduced nicotinamide adenine dinucleotide; NADP+, oxidized nicotinamide adenine dinucleotide‐phosphate; NADPH, reduced nicotinamide adenine dinucleotide‐phosphate. (1) Pyruvate dehydrogenase complex. (2) α‐Ketoglutarate dehydrogenase complex. (3) Transketolase. Created in BioRender. Rosewarne, R. (2025) https://BioRender.com/6emaoq3. Adapted from: Frank LL (6).

Thiamine exists in both a free and phosphorylated form (known as thiamine pyrophosphate or TPP) [7]. TPP is biologically active and essential in carbohydrate metabolism, neural function, and metabolism of glyoxylate (Figure 1) [6]. Under normal conditions, pyruvate produced from glycolysis is converted to acetyl coenzyme A by pyruvate dehydrogenase, which is then utilized within the Krebs cycle for adenosine triphosphate production. However, in the absence of sufficient oxygen, pyruvate is shunted to the lactic acid cycle where it is reduced to lactate [6]. Additionally, thiamine deficiency also shunts pyruvate to the lactic acid cycle [6]. This is due to thiamine serving as a cofactor for the conversion of pyruvate to acetyl coenzyme A. Thiamine also serves as a coenzyme for transketolase in the pentose phosphate pathway. This pathway is essential to produce reduced nicotinamide adenine dinucleotide‐phosphate (NADPH), which is used for many functions within the body like synthesis of fatty acids, maintenance of myelin sheaths, nerve membrane function, and transmission [6].

Similar to many medications, absorption of thiamine primarily occurs within the small intestine. At low concentrations (< 2 μM), absorption is primarily an active, carrier‐mediated, saturable process with greatest activity in the duodenum [7]. At high concentrations (> 2.5 mg), absorption occurs through passive diffusion [7]. A study published in 2012 evaluated the pharmacokinetics of multiple oral thiamine dosing regimens in healthy patients. Each patient received placebo, 100 mg of thiamine, 500 mg of thiamine, and 1500 mg of thiamine with a minimum of 1 week elapsing between trials. Whole blood and plasma concentrations were evaluated at hour 0.5, 1, 1.5, 2, 3, 4, 5, 6, 8, and 10 following each dose of study medication. The authors found that not only was the overall effect of the dose significant (plasma: *p* < 0.001 and whole blood *p* < 0.001), the mean AUC_0–10 hr_ for each dose was also significantly different (*p* < 0.05) [8]. This study demonstrated that thiamine absorption is not saturable up to 1500 mg and that administration of higher doses of oral thiamine is associated with higher overall patient exposure.

Thiamine deficiency can be difficult to diagnose due to its associated nonspecific symptoms. Symptoms of thiamine deficiency include ataxia, ocular dysfunction, altered mental status, tachycardia, and/or peripheral edema [9]. Deficiency can develop for a variety of reasons including short bowel syndrome, severe malnutrition, alcohol use disorder, or diuretic use. Each mechanism of deficiency is unique but can be described as a disruption in either intake, absorption, or excretion. Patients with short bowel syndrome can experience a decreased absorption of thiamine following surgery due to damage to the small intestine or either partial or complete removal of the small intestine. Alcohol use disorder impacts the absorption of thiamine. Ethanol inhibits the active transport of thiamine through inhibition of human thiamine transporter‐1 gene expression in our intestinal cells [10]. This prevents thiamine from being absorbed into the bloodstream. Alcohol alone can decrease absorption by ∼50% in healthy patients [11]. When coupled with malnutrition, thiamine absorption is decreased to 70% [11]. Thiamine and its metabolites are primarily excreted in the urine. In patients taking loop diuretics, thiamine excretion has been observed to double compared to patients not taking diuretics [12].

Suspected or confirmed thiamine deficiency is primarily treated through supplementation of thiamine. Supplementation is often well‐tolerated with minimal reports of adverse effects. High doses of oral thiamine have been most commonly associated with nausea, though specific dosing associated with “high dose” has not been well described. Intravenous and intramuscular thiamine have been associated with injection site reactions, flushing, and hypersensitivity reactions. Regardless of formulation, thiamine is inexpensive and often easily obtainable.

Thiamine supplementation has also been investigated for many indications such as Wernicke’s encephalopathy (WE), refeeding syndrome, and sepsis. WE and refeeding syndrome are two indications that have established benefit for thiamine supplementation. Each has published guidelines to aid providers in treating these unique populations; however, there are differences in dosing recommendations in the guidelines leading to inconsistencies in the selection of thiamine dose, frequency, and duration of therapy. Additionally, recent evidence for additional uses of thiamine—such as sepsis, lactate clearance, and delirium—has emerged with conflicting or sparse evidence. Prior summaries have focused on the emerging areas for thiamine use in critical illness and evaluation of relevant studies. The purpose of this narrative review is to provide an overview of the historical and emerging indications for thiamine supplementation in hospitalized patients, analyze literature for thiamine supplementation utilization, and provide a framework for supplementation based on indication.

## 2. Established Uses for Thiamine

### 2.1. WE

WE is a severe neurologic complication resulting from thiamine deficiency often incited by alcohol consumption [13]. It is characterized by a triad of symptoms: altered mental status, ataxia, and ocular dysfunction [14]. Due to its generalizable symptoms and lack of diagnostic test, WE is primarily diagnosed through exclusion of other probable diagnoses. Alternative diagnoses to be considered are hepatic encephalopathy, delirium, stroke, alcohol intolerance, and medication‐related side effects. Once Wernicke’s is suspected, prompt initiation of thiamine supplementation is recommended along with symptom management. Symptoms often begin to resolve within 24–48 h of thiamine supplementation initiation [15–18].

There are many case reports describing successful treatment of WE. However, there is significant variation in the thiamine route of administration and dosing when comparing cases. One case report described a 66‐year‐old male who was diagnosed with nonalcohol‐induced WE 14 days following surgery for acute necrotic–hemorrhagic pancreatitis. The patient was treated with 100 mg of intravenous thiamine once daily for 4 days. Over the course of treatment, all neurologic signs and symptoms gradually resolved [15]. A second case report described a 31‐year‐old female, with a history of Roux‐en‐Y gastric bypass, who was diagnosed with alcohol‐induced WE. The patient was treated with 500 mg of intravenous thiamine three times a day for 2 days followed by 500 mg of intravenous thiamine daily for 7 days. While the time from treatment initiation to resolution of symptoms was not described, they were noted to have improved at a 6‐week follow‐up visit [16]. An additional case report described a 47‐year‐old male, with a history of severe Crohn disease, who was diagnosed with nonalcohol‐induced WE following surgery for an elective colectomy, which was complicated by an enterocutaneous fistula. The patient was treated with 200 mg of intravenous thiamine three times a day for an unspecified duration. Following 24 h of supplementation, the patient’s condition began to improve [17]. Lastly, a case report described a 20‐year‐old female who was diagnosed with nonalcohol‐induced WE three months following sleeve gastrectomy. The patient was treated with one dose of 500 mg of intravenous thiamine followed by 100 mg of intravenous thiamine day for an unspecified duration. While the timeframe of acute resolution was not described, nystagmus and dysarthria were noted to have improved throughout her hospital course [18]. Each of these four case reports describes successful treatment of Wernicke’s; however, there is lack of consistency in the treatment regimens.

There are several guidelines to aid clinicians in the selection of thiamine dose, frequency, and duration of therapy (Table 2). The 2010 European Journal of Neurology recommend that patients with suspected or confirmed WE receive intravenous thiamine 200 mg three times daily until there is no further improvement in signs and symptoms. In patients at risk for thiamine deficiency, it was recommended that prophylactic intravenous thiamine be started at 200 mg daily for an unspecified duration [19]. The 2012 British Association for Psychopharmacology guidelines for the pharmacological management of substance abuse, harmful use, addiction, and comorbidity recommend that patients with suspected or confirmed WE receive 500 mg of parenteral thiamine three times daily for 3–5 days followed by parenteral thiamine 250 mg once daily for 3–5 days based on response. For patients at high risk for developing WE, these guidelines also recommend that prophylactic parenteral thiamine be started at 250 mg daily for 3–5 days or until no further improvement is seen. In healthy patients with uncomplicated alcohol use undergoing detoxification, it was recommended that daily oral thiamine be initiated at 300 mg or higher and continued throughout the detoxification process [20]. The most recent guidelines addressing WE were published in 2021 from the University of Sydney. These guidelines recommend that patients with suspected or confirmed WE receive parenteral thiamine 500 mg per day for 3–5 days followed by 300 mg–1000 mg per day, either enterally or parenterally, for 1–2 weeks. Prophylactic parenteral thiamine 300 mg per day for 3–5 days followed by enteral thiamine 300 mg per day for several weeks was recommended for patients with chronic alcohol use with poor nutritional status [21].

**TABLE 2:** Guideline recommendations for prevention and treatment of Wernicke’s encephalopathy.

Guideline, year published	Initial treatment regiment	Strength of recommendation	Step‐down treatment regimen	Strength of recommendation	Prevention	Strength of recommendation
European Journal of Neurology, 2010	IV thiamine 200 mg TID until there is no further improvement in signs and symptoms	Possibly effective, ineffective, or harmful	—	—	IV thiamine 200 mg daily for unspecified duration	GPP
British Association for Psychopharmacology, 2012	IV/IM thiamine 500 mg TID for 3–5 days	Directly based on category IV evidence or extrapolated recommendation from category I, II, or III evidence	IV/IM thiamine 250 mg once daily for 3–5 days based on response	Directly based on category IV evidence or extrapolated recommendation from category I, II, or III evidence	IV/IM thiamine 250 mg daily for 3–5 days or until no further improvement is seen	Directly based on category IV evidence or extrapolated recommendation from category I, II, or III evidence
University of Sydney, 2021	IV/IM thiamine 500 mg per day for 3–5 days	GPP	PO/IV/IM thiamine 300–1000 mg per day for 1–2 weeks	Body of evidence is weak, and recommendation must be applied with caution	IV/IM thiamine 300 mg per day for 3–5 days followed by PO thiamine 300 mg per day for several weeks	Body of evidence is weak, and recommendation must be applied with caution

*Note:* Definitions: category IV evidence, evidence from expert committee reports or opinions and/or clinical experience of respected authorities; category I evidence, evidence from meta‐analysis of randomized controlled trials or at least one randomized controlled trial; category II evidence, evidence from at least one controlled study without randomization or one other type of quasi‐experimental study; category III evidence, evidence from nonexperimental descriptive studies, such as comparative studies, correlation studies, and case–control studies.

Abbreviations: GPP, good practice point; IM, intramuscular; IV, intravenous; PO, oral; TID, three times daily.

Variability among recommendations published in the available guidelines can largely be attributed to the age of publication, practice pattern of the specific region represented, and product availability. While there is variability among available guidelines, it is generally recommended that patients with suspected or confirmed WE receive at least 500 mg of parenteral thiamine per day for 3–5 days followed by a step‐down regimen for several days [19–21]. Each of the guidelines agrees that patients at risk for developing Wernicke’s should receive parenteral thiamine 200–300 mg daily for 3–5 days [19–21]. If patients do not have parenteral access and thiamine supplementation is desired, enteral thiamine can be administered at doses similar to recommended parenteral doses.

As previously mentioned, WE symptoms should begin to resolve within 24–48 h of thiamine initiation. If the patient demonstrates no improvement at that time, alternate causes of symptoms should be investigated, and discontinuation of thiamine should be considered.

### 2.2. Refeeding Syndrome

Refeeding syndrome is the metabolic and electrolyte alterations resulting from the reintroduction and/or increased nutrition after a period of substantially decreased or absent caloric intake. Due to poor nutritional intake, patients at risk for developing refeeding syndrome are likely to also have an underlying thiamine deficiency. Reintroduction of nutrition also increases thiamine demand (Figure 1) [6, 22]. As a result, patients at risk for refeeding syndrome may develop symptoms of thiamine deficiency when nutrition is reintroduced. These symptoms would likely present similar to patients with WE. However, symptomatic exacerbation of thiamine deficiency when nutrition is reintroduced is poorly described in the literature and the incidence is not well known. Nevertheless, thiamine is often supplemented in patients at risk for refeeding syndrome to prevent exacerbation of thiamine deficiency. Thiamine supplementation, however, does not prevent or treat the electrolyte abnormalities that may develop during refeeding syndrome.

The American Society of Parenteral and Enteral Nutrition (ASPEN) and the European Society for Clinical Nutrition and Metabolism (ESPEN) provide recommendations for supplementation in patients at risk for developing refeeding syndrome (Table 3) [22, 23]. Both societies recommend the administration of thiamine prior to the administration of intravenous glucose [22, 23]. This is due to the theorized decreased absorption of glucose in the setting of thiamine deficiency and risk of exacerbating thiamine deficiency when glucose is introduced. However, there is a lack of evidence to support this practice routinely. Some guidelines are beginning to remove this recommendation, stating that it is not necessary to administer thiamine prior to glucose in order to prevent delays in treatment [24].

**TABLE 3:** Society recommendations for prevention and treatment of refeeding syndrome.

	ASPEN 2020 consensus recommendations	Grade of recommendation	ESPEN 2022 guideline	Grade of recommendation
Levels	Thiamine levels should not be obtained for emergency management but may be useful for subsequent care.	Not Available	RBC or whole blood thiamine diphosphate should be collected though treatment should not be based on result. Result is used to confirm diagnosis.	A^2^
Initial Supplementation				
At risk patients	Thiamine 100 mg once before feeding or before initiating dextrose‐containing IV fluids	Not Available	Thiamine 100 mg three times a day	B^3^
High‐risk patients^1^	Thiamine 100 mg per day for 5–7 days or longer	Not Available	Thiamine 200 mg three times a day	B^3^
Continued Supplementation				
Parenteral nutrition	MVI should be continued daily for duration of PN therapy	Not Available	Should provide at least 2.5 mg per day of thiamine	B^3^
Oral/enteral nutrition	A daily oral/enteral multivitamin should be added for at least 10 days or greater	Not Available	Should provide 1.5–3 mg per day of thiamine in patients receiving 1500 kcal per day.	A^2^

Abbreviations: ASPEN, American Society for Parenteral and Enteral Nutrition; ESPEN, European Society for Clinical Nutrition and Metabolism

^1^Severe starvation, chronic alcoholism, or other high risk for deficiency and/or signs of thiamin deficiency.

^2^Grade A: At least one high‐quality meta‐analysis, systematic review, or RCT rated as 1++ and directly applicable to the target population or a systematic review of well‐conducted RCTs or a body of evidence consisting principally of well‐conducted studies directly applicable to the target population and demonstrating overall consistency of results.

^3^Grade B: At least one high‐quality meta‐analysis, systematic review, or RCT rated as 1++ and directly applicable to the target population or a systematic review of well‐conducted RCTs or a body of evidence consisting principally of well‐conducted studies directly applicable to the target population and demonstrating overall consistency of results.

In patients at risk of developing refeeding syndrome, thiamine supplementation should be initiated to prevent further exacerbation of potential thiamine deficiency. ASPEN guidelines recommend 100 mg of thiamine once daily for 5–7 days but do not specify the route of administration. ESPEN guidelines recommend 200 mg of intravenous thiamine three times daily. Variability among the ASPEN and ESPEN guideline recommendations is common and not unique to refeeding syndrome. This is largely a result of differences in the methodology of evidence interpretation and age of publication. Based on both guideline recommendations, thiamine at a dose of at least 100 mg daily for 7 days should be initiated either enterally or parenterally when nutrition is resumed. In patients who are asymptomatic or have mild symptoms of thiamine deficiency (such as fatigue, irritability, poor memory, sleep disturbances, etc.), oral thiamine supplementation is appropriate [22]. If oral access is not available, parenteral thiamine is an appropriate alternative. Following this period of supplementation, patients receiving parenteral nutrition should have multivitamin infusion included within their parenteral nutrition prescription daily for the duration of parenteral nutrition therapy. In patients with moderate to severe symptoms of thiamine deficiency (such as altered mental status, ataxia, ocular dysfunction, etc.), WE should be suspected and additional parenteral thiamine supplementation should be initiated.

## 3. Emerging Uses for Thiamine

### 3.1. Sepsis

Sepsis is a life‐threatening organ dysfunction caused by a dysregulated response to infection [25]. Sepsis has a significant impact on the healthcare system with at least 1.7 million adults in the U.S. developing sepsis in 2024 and at least 350,000 dying as a result [26]. Currently, sepsis is the leading cause of death among hospitalized patients and the leading cause for hospital readmissions [26]. In 2021, the aggregate hospital cost for all sepsis‐related stays reached $51.2 billion [27]. Because of the impact sepsis has on health systems, identifying interventions that improve patient outcomes has been a priority among healthcare providers.

A retrospective before–after study published in 2016 evaluated the impact of thiamine, ascorbic acid (vitamin C), and hydrocortisone on hospital survival in patients with severe sepsis or septic shock. The investigators included thiamine in their treatment intervention due to thiamine deficiency being associated with an increased risk of death in patients with septic shock. This was a single‐center analysis that compared 47 patients who received a sepsis treatment protocol consisting of intravenous thiamine, hydrocortisone, and ascorbic acid to 47 historical control patients who did not receive this bundle. The authors found that administration of ascorbic acid, thiamine, and hydrocortisone was associated with much lower hospital mortality (8.5% vs. 40.4%; *p* < 0.001) [28]. This study yielded promising results that had the potential to change the approach to sepsis management. However, the results were viewed skeptically by many in the sepsis community due to their extreme nature and many randomized controlled trials were subsequently conducted in an effort to validate these results.

These validation trials spanned many years and received substantial amounts of funding. While each validation trial had variable methodology, each failed to demonstrate that the thiamine, ascorbic acid, and hydrocortisone bundle improved mortality when compared to placebo (Table 4) [29–36]. The importance of validation from randomized, controlled trials in the face of disproportional results from uncontrolled trials served as a reminder. While uncontrolled trials are beneficial for hypothesis generation, large practice patterns should not be easily swayed by their results until validation is complete.

**TABLE 4:** Summary of studies evaluating thiamine use in sepsis.

Author, year published	Intervention	Comparator	Mortality outcome(s)	Other notable outcomes
Fujii, 2020	• IV vitamin C 1.5 g every 6 h • IV hydrocortisone 50 mg every 6 h • IV thiamine 200 mg every 12 h	• IV hydrocortisone 50 mg every 6 h	• 28‐day mortality: 22.6% vs. 20.4% (95% CI: −8.9%–13.4%; *p* = 0.69) • 90‐day mortality: 28.6% vs. 24.5% (95% CI: −8.0%–16.1%; *p* = 0.51)	• Time alive and free of vasopressors at day 7 after randomization: 122.1 h vs 124.6 h (*p* = 0.83)

Iglesias, 2020	• IV vitamin C 1.5 g every 6 h • IV hydrocortisone 50 mg every 6 h • IV thiamine 200 mg every 12 h	Placebo	• Hospital mortality: 16% vs. 19.4% (odds ratio: 1.2; 95% CI: 0.50–2.97; *p* = 0.6)	• Change in the SOFA score between enrollment and 72‐h follow‐up: 2.9 vs. 1.93 (*p* = 0.17) • Duration of vasopressors: 27 h vs. 53 h (*p* < 0.001)

Chang, 2020	• IV vitamin C 1.5 g every 6 h • IV hydrocortisone 50 mg every 6 h • IV thiamine 200 mg every 12 h	Placebo	• 28‐day mortality: 27.5% vs. 35% (*p* = 0.47)	• Change in the SOFA score between enrollment and 72‐h follow‐up: 3.5 vs. 1.8 (*p* = 0.02)

Moskowitz, 2020	• IV vitamin C 1.5 g every 6 h • IV hydrocortisone 50 mg every 6 h • IV thiamine 100 mg every 6 h	Placebo	• 30‐day mortality: 34.7% vs. 29.3% (hazard ratio, 1.3; 95% CI, 0.8–2.2; *p* = 0.26)	• Change in the SOFA score between enrollment and 72‐h follow‐up: Mean difference, −0.8 (95% CI, −1.7–0.2; *p* = 0.12)

Hwang, 2020	• IV vitamin C 50 mg/kg (max single dose 3 g, daily dose 6 g) every 12 h • IV thiamine 200 mg every 12 h	Placebo	• 7‐day mortality: 9.4% vs. 10.3% (*p* = 0.87) • 28‐day mortality: 20.8% vs. 15.5% (*p* = 0.47) • 90‐day mortality: 32.1% vs. 27.6% (*p* = 0.61)	• Change in the SOFA score between enrollment and 72‐h follow‐up: 3 vs. 3 (*p* = 0.96)

Sevransky, 2021	• IV vitamin C 1.5 g every 6 h • IV hydrocortisone 50 mg every 6 h • IV thiamine 100 mg every 6 h	Placebo	• 180‐day mortality: 40.5% vs. 37.8% (difference 2.7; 95% CI: −11.3–5.8; *p* = 0.53)	• Ventilator‐ and vasopressor‐free days in the first 30 days following randomization: 25 days vs. 26 days (median difference: −1 day; 95% CI, −4 to 2 days; *p* = 0.85)

Mohamed, 2023	• IV vitamin C 1.5 g every 6 h • IV hydrocortisone 50 mg every 6 h • IV thiamine 200 mg every 12 h	Standard of care only	• 60‐day hospital mortality: 28.3% vs. 35.8% (*p* = 0.41)	• Duration of vasopressor therapy: 50 h vs. 58 h (*p* = 0.44) • Change in the SOFA score between enrollment and 72‐h follow‐up: −1 vs. −2 (*p* = 0.54)

Pereira, 2023	• IV thiamine 200 mg every 12 h	Placebo	• 28‐day mortality: 56.1% vs. 60.3% (*p* = 0.789)	• Vasoactive drug‐free days: 9 days vs. 5 days (*p* = 0.342)

Sepsis remains a common and severe syndrome impacting patients across the globe [21]. Future research should focus on identifying evidence‐based interventions to improve outcomes in these patients.

### 3.2. Lactate Clearance

Lactate is a byproduct of anaerobic metabolism. Increased lactate can occur for a variety of reasons such as strenuous exercise, use of certain medications, seizures, blood loss, and sepsis [8]. Severe hyperlactatemia may result in lactic acidosis. In patients with sepsis and septic shock, elevated initial lactate levels have been associated with an increased risk of all‐cause mortality [37]. Acute acidosis impairs myocardial contractility, reduces cardiac output, causes arterial vasodilation with hypotension, predisposes to arrhythmias, impairs oxygen delivery, decreases adenosine triphosphate production, and suppresses immune responses [38]. Therefore, timely treatment of acute acidosis is important to decrease morbidity and mortality. It is theorized that thiamine supplementation in patients with a lactic acidosis may aid in normalization of lactate levels. Thus, thiamine supplementation may be particularly helpful for lactate clearance in patients with baseline thiamine deficiency.

There are a few studies examining thiamine use for lactate clearance, particularly among patients diagnosed with septic shock. One randomized, double‐blind study published in 2016 evaluated the impact of parenteral thiamine on lactate reduction in patients diagnosed with septic shock. Patients received either 200 mg of parenteral thiamine or placebo twice daily for 7 days or until hospital discharge. Thiamine was not associated with a difference in overall median lactate levels at 24 h (2.5 mmol/L vs. 2.6 mmol/L; *p* = 0.40); however, in the repeated measures model, there were lower lactate levels in the thiamine group at 24 h (*p* = 0.048). Of the 92 patients included in this study, 79 patients had baseline thiamine levels measured and 35% were found to have thiamine deficiency. Among the patients with a baseline thiamine deficiency, those who received thiamine had lower median lactate levels at 24 h (2.1 mmol/L vs. 3.1; *p* = 0.03) as well as lower lactate levels in the thiamine group at 24 h in the repeated measures model (*p* = 0.006) [39]. A second retrospective, single‐center study published in 2018 evaluated the impact of intravenous thiamine on time to lactate clearance in patients diagnosed with septic shock. Thiamine was administered upon clinician suspicion of thiamine deficiency instead of for the treatment of septic shock. The majority of patients received 500 mg (range: 100–500 mg) of thiamine for a median duration of 66 h. In this study, thiamine supplementation was associated with improved lactate clearance (subdistribution hazard ratio: 1.339; 95% confidence interval [CI]: 1.044–1.717) and reduction in 28‐day mortality (hazard ratio: 0.666; 95% CI: 0.490–0.905) [40]. A third randomized, double‐blind study published in 2020 evaluated the impact of parenteral thiamine on vasopressor requirements in patients diagnosed with septic shock. Patients received either 200 mg of intravenous thiamine or placebo twice daily for 7 days or until hospital discharge. In this study, thiamine supplementation was associated with a greater median reduction in lactate at 24 h compared to those in the placebo group (1.0 mmol/L vs. 0.5 mmol/L; *p* = 0.024) [41].

Despite the lower sample sizes found in these studies, they demonstrate a potential role of thiamine for lactate clearance. Thiamine can be considered for lactate clearance in patients with sepsis who have concomitant lactic acidosis with lactate not responding to appropriate fluid resuscitation and antibiotic initiation. Current available literature suggests that intravenous thiamine of 200–500 mg as a one‐time dose can be administered in these patients [39–41]. Future research should focus on the duration of therapy. A one‐time dose of thiamine may be sufficient to normalize lactate levels. Additionally, current literature largely focuses on lactate clearance in patients with septic shock; however, future studies should seek to understand if thiamine may be beneficial for lactate clearance when accumulation occurs in the absence of sepsis.

### 3.3. Delirium

Delirium is highly prevalent in critically ill patients, particularly among those receiving mechanical ventilation [42]. It is characterized by an acute alteration of attention, consciousness, and cognition [43]. Delirium has been associated with worsened clinical outcomes including mortality, prolonged hospital length of stay, and worse long‐term cognitive function with poorer trajectories seen in those with prolonged durations of delirium [43]. There are many gaps of literature including how to prevent the development of delirium and how to treat it once it has developed. Current guideline recommendations focus largely on nonpharmacologic prevention and treatment of delirium [44, 45].

Thiamine deficiency has been proposed as a contributor to delirium development. As previously discussed, thiamine serves as a coenzyme for transketolase in the pentose phosphate pathway that produces NADPH, which is responsible for a variety of functions including the production of glutathione. Glutathione is an antioxidant that forms the first line of defense against reactive oxygen species‐induced oxidative stress [46, 47]. Glutathione peroxidase converts hydrogen peroxide (H_2_O_2_) to 2 H_2_O using reduced glutathione as a substrate [46, 47]. This process oxidizes glutathione, which is then converted back to its reduced form using the glutathione reductase and NADPH [46, 47]. Impairment of this process leads to excess H_2_O_2_ converting to hydroxyl free radicals causing cellular injuries in neuronal cells, which can present as delirium [46].

A systemic review and meta‐analysis that was published in 2021 evaluated the use of thiamine in critical illness. Of the 18 studies (8 randomized control trials [RCTs] and 10 cohort studies) included in the meta‐analysis, 4 studies (3 RCTs and 1 cohort; *n* = 497) reported the incidence of delirium. The odds ratio (OR) for the incidence of delirium among the thiamine supplemented group compared with that of the placebo/standard‐of‐care group was 0.78 (95% CI: 0.52–1.15; I2 = 35%). When the one cohort study was excluded, the authors found a 42% lower odds of developing ICU delirium (OR 0.58; 95% CI: 0.34–0.98; *n* = 319; I2 = 0%) among the group that received intravenous thiamine [48]. However, the RCTs included in this meta‐analysis had large variations in the populations that were studied. The first RCT was published in 2016 evaluating the effect of intravenous thiamine on postoperative lactate levels in patients undergoing coronary artery bypass grafting [49]. Delirium following cardiac surgery is common due to the use of an intraoperative cardiopulmonary bypass among other patient‐specific risk factors like age and past medical history [50]. The second RCT was published in 2020 evaluating the effect of intravenous ascorbic acid, hydrocortisone, and thiamine on change in the SOFA in patients with sepsis [32]. In this study, there were multiple confounding variables making it difficult to determine if thiamine supplementation had an impact on delirium development. The third RCT was published in 2020, evaluating the effect of intravenous thiamine on postoperative delirium in patients undergoing gastrointestinal surgery in Iran [51]. Findings from this trial may be difficult to extrapolate to nonsurgical patients or other surgical populations. The differences in patient populations between each of the studies make it difficult to interpret the results found in the meta‐analysis [48].

Thiamine supplementation for the prevention and/or treatment of delirium is an emerging area of research. Current available literature evaluating the use of thiamine for delirium prevention is limited, and existing delirium guidelines make no recommendations for thiamine use. Further research is needed before thiamine can be routinely recommended to prevent delirium.

## 4. Conclusion

Thiamine is an essential vitamin in many human metabolic pathways. Current evidence strongly supports thiamine supplementation for the treatment of WE and for the prevention of symptomatic exacerbation of thiamine deficiency in patients with baseline malnutrition. Table 5 provides a summary of thiamine supplementation recommendations. Thiamine deficiency can result in severe consequences such as WE, necessitating prompt thiamine supplementation. Patients with baseline malnutrition may have thiamine deficiency as a result, and thiamine supplementation should be initiated as soon as possible when reintroducing enteral or parenteral nutrition.

**TABLE 5:** Summary table for thiamine supplementation recommendations.

Indication	Dose	Route	Duration
Established Uses			
Suspected or Confirmed Wernicke’s Encephalopathy	500 mg	Parenteral	3–5 days
At Risk of Wernicke’s Encephalopathy	200–300 mg	Enteral or Parenteral	3–5 days
Refeeding Syndrome	100 mg	Enteral or Parenteral	7 days
Emerging Uses			
Sepsis	Not Recommended		
Lactate Clearance	200–500 mg	Parenteral	One‐time dose
Delirium	Further Research Needed		

In recent years, many studies have been published evaluating the use of thiamine for mortality reduction in sepsis. The majority of studies show that thiamine does not reduce mortality in sepsis and should not be used for this indication. However, the use of thiamine may aid in lactate clearance in patients with refractory lactic acidosis. Similarly, the benefit of thiamine for delirium prevention is uncertain. Future research initiatives should look to clarify optimal dose and duration of therapy for thiamine use in syndromes seen in the intensive care unit, including sepsis and delirium.

## Funding

No funding was received for this manuscript.

## Conflicts of Interest

The authors declare no conflicts of interest.

## Data Availability

Data sharing is not applicable to this article as no datasets were generated or analyzed during the current study.
